# Genetic and Environmental Influences on Motor Function: A Magnetoencephalographic Study of Twins

**DOI:** 10.3389/fnhum.2014.00455

**Published:** 2014-06-19

**Authors:** Toshihiko Araki, Masayuki Hirata, Hisato Sugata, Takufumi Yanagisawa, Mai Onishi, Yoshiyuki Watanabe, Kayoko Omura, Chika Honda, Kazuo Hayakawa, Shiro Yorifuji

**Affiliations:** ^1^Division of Functional Diagnostic Science, Osaka University Medical School, Suita, Japan; ^2^Department of Neurosurgery, Osaka University Medical School, Suita, Japan; ^3^Department of Diagnostic and Interventional Radiology, Osaka University Medical School, Suita, Japan; ^4^Center for Twin Research, Osaka University Medical School, Suita, Japan

**Keywords:** twins, motor function, movement-related cortical fields, magnetoencephalography

## Abstract

To investigate the effect of genetic and environmental influences on cerebral motor function, we determined similarities and differences of movement-related cortical fields (MRCFs) in middle-aged and elderly monozygotic (MZ) twins. MRCFs were measured using a 160-channel magnetoencephalogram system when MZ twins were instructed to repeat lifting of the right index finger. We compared latency, amplitude, dipole location, and dipole intensity of movement-evoked field 1 (MEF1) between 16 MZ twins and 16 pairs of genetically unrelated pairs. Differences in latency and dipole location between MZ twins were significantly less than those between unrelated age-matched pairs. However, amplitude and dipole intensity were not significantly different. These results suggest that the latency and dipole location of MEF1 are determined early in life by genetic and early common environmental factors, whereas amplitude and dipole intensity are influenced by long-term environmental factors. Improved understanding of genetic and environmental factors that influence cerebral motor function may contribute to evaluation and improvement for individual motor function.

## Introduction

Human motor function is attributed by both innate and acquired traits (Lippi et al., 2010; Tucker and Collins, 2012). Innate traits are mainly related to genetic factors, whereas acquired traits are related to environmental factors. Accordingly, although motor function changes with environmental influences such as training, certain genes have been associated with motor function. In particular, the angiotensin converting enzyme (ACE) I allele and the ACTN3 gene (encoding alpha-actinin-3) have been associated with athletic excellence (Gayagay et al., 1998; Yang et al., 2003). Thus in the present study, we hypothesized that cerebral motor function is affected by genetic and environmental influences, based on the assumption that genetically identical brains function differently following long-term exposure to different environments, and reflect brain plasticity. It is important for sports education and training to clarify it. However, little is known to what extent environmental and genetic factors influence cerebral motor function. Middle-aged and elderly monozygotic (MZ) twins are good subjects to investigate the environmental and genetic effects on brain function because a middle-aged and elderly MZ pair is identical with regards to genetic factors but different regarding their environmental exposure. Many studies have compared similarities in electrical brain activity between MZ twins and dizygotic (DZ) twins using electroencephalogram (EEG) and magnetoencephalogram (MEG) (van Beijsterveldt and van Baal, 2002; Smit et al., 2005; Begleiter and Porjesz, 2006; van Pelt et al., 2012). For example, using EEG, the genetic effects on amplitudes and waveforms of several evoked potentials were reported by comparing MZ twins with DZ twins or unrelated pairs (Lewis et al., 1972). Recently, Van’t Ent et al. (2010) revealed genetic influences on waveform amplitude and morphology of entire time series of somatosensory-evoked brain activity in a sample of MZ and DZ twins. However, published twin studies concerning brain activity were limited to primary sensory functions such as visual, auditory, and somatosensory-evoked potentials, and there are no reports to this date on motor-related brain function.

Using MEG, typical responses are observed during voluntary finger movement called movement-related cortical fields (MRCFs) (Cheyne and Weinberg, 1989). MRCFs are representative cortical responses for evaluation of motor function. For example, abnormalities of the time course of MRCFs have been associated with movement disorders such as Parkinson’s disease and dystonia (Chen and Hallett, 1999). MRCFs consist of several components, one of which is movement-evoked field 1 (MEF1), which shows the largest and most robust signal (Kristeva et al., 1991; Kristeva-Feige et al., 1995). MEF1 appears approximately 80–120 ms after the onset of muscle contraction (Cheyne and Weinberg, 1989; Cheyne et al., 1997), and the current source underlying MEF1 has been located in primary somatosensory cortex (Cheyne et al., 1997, 2006; Onishi et al., 2006) or primary motor cortex (Onishi et al., 2011). Moreover, peak amplitude of MEF1 increased in Tourette syndrome patients who were characterized by motor tics (Biermann-Ruben et al., 2012), it is considered one of the most important components of MRCFs produced during voluntary movement. In this study, we focused on MEF1 to investigate how genetic and environmental factors affect movement-related brain activity by evaluating differences between middle-aged and elderly MZ twins and unrelated age-matched pairs.

## Materials and Methods

### Subjects

Monozygotic or DZ twins of ≥20 years of age and with no history of neurological or psychiatric episodes were recruited by the Center for Twin Research in Osaka University. We assessed 16 healthy pairs of MZ twins and 15 unrelated individuals (23 males: 65.5 ± 9.5 years; 22 females: 60.3 ± 10.9 years). All subjects were determined to be right-handed by a score in the Edinburgh inventory. Zygosity was determined by short tandem repeat (STR) typing. Written informed consent was obtained from all subjects after explanation of the purpose and possible consequences of the study. This study was approved by the ethics committee of the Osaka University Graduate School of Medicine.

Osaka University Center for Twin Research was organized to collect various information as well as biological resources from registered twins, and to establish a biobank and databases for preserving and managing these data and resources (Hayakawa et al., 2013). The following data are being collected: physical data (e.g., height, body mass, and bone density), data regarding epidemiology (e.g., medical history, lifestyle, cognitive function, and nutrition), EEG, ultrasonography, dentistry, plastic surgery, positron emission tomography, MEG, and magnetic resonance imaging (MRI). In this research, we investigated the cerebral motor function using MEG.

### Measurements

The subjects lay on a bed comfortably in the supine position with their head centered into the gantry. They were instructed to close their eyes and to lift the right index finger at self-paced intervals of approximately 5 s. Movements were performed with a very sharp onset, and started from total muscular relaxation. Neuromagnetic activities were recorded in a magnetically shielded room using a 160-channel whole-head MEG system equipped with coaxial type gradiometers (MEG vision NEO; Yokogawa Electric Corporation, Kanazawa, Japan). Data were acquired at a rate of 1000 Hz with an online low-pass filter at 200 Hz. The positions of five head marker coils were obtained before and after recording to localize head position and to evaluate head movement. The maximum acceptable head movement was set at 5 mm and head movements ranged from 0.03 to 3.53 ms. Because head movements were small, the head movement compensation algorithm was not required. Anatomical MRI data were obtained using a 3.0-T magnetic resonance scanner with a standard whole-head coil (Signa HDxt Excite 3.0 T, GE Healthcare UK Ltd., Buckinghamshire, UK).

In order to align MEG data with individual MRI data, we scanned the three-dimensional facial surface of each participant (FastSCAN Cobra, Polhemus, USA). Five head marker coils were attached to the scalp before recording MEG, which provided the position and orientation of MEG sensors relative to the head. Three-dimensional facial surface data were superimposed on the anatomical facial surface provided by the MRI data. We also recorded electromyograms of the right extensor indicis muscle and monitored the self-paced movement of the subjects using two video cameras.

### Data analyses

**Figure 1:**
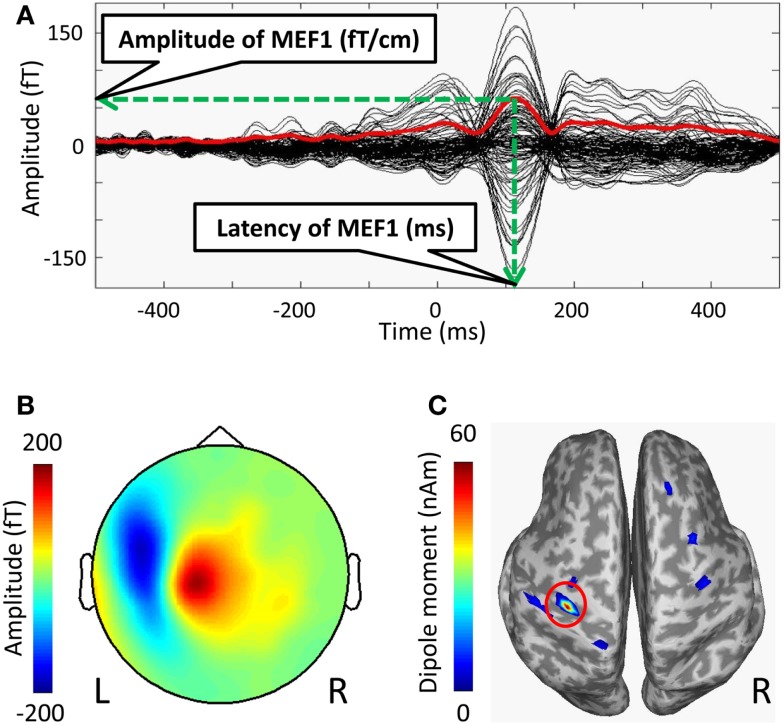
**Averaged waveforms, a topographic map, and estimated current sources at MEF1 of the MRCFs in one subject**. **(A)** Time average of MRCFs for 125 channels (black lines) and the root mean square (RMS) waveforms calculated from 125 channels (red line). The peak amplitude of RMS waveforms in MEF1 was defined as the amplitude of MEF1, and the peak latency was defined as the latency of MEF1. **(B)** The topographic map (nose upwards) at the peak latency of MEF1 (red: outflow, blue: inflow). **(C)** The amplitude map of estimated current source at the latency of MEF1. The activity map was rendered on inflated cortical surface. Sulci (concave) and gyri (convex) were indicated by dark and light-gray shading, respectively. The coordinates (*x*, *y*, *z*) with the maximum dipole moment in the MNI reference brain were defined as the dipole location of MEF1 (red circle), and the dipole moment value was defined as the dipole intensity of MEF1.

The MEG data were analyzed using the standard MEG software of the system (MEG Laboratory; Yokogawa Electric Corporation, Kanazawa, Japan). The onset of each self-paced finger movement was manually determined by an initial rise in the electromyogram waveforms, and this onset time was defined as 0 ms. The time-window of each recoding epochs were defined from −500 to 500 ms. Epochs containing artifacts, such as obvious eye movements and excessive muscle activity were eliminated from the analyses. The epochs were averaged using from −500 to −300 ms as a baseline. Averaged waveforms were high-pass filtered using a cut-off frequency of 1 Hz, and low-pass filtered using a cut-off frequency of 20 Hz. We excluded 35 channels of the bilateral frontal base from the analyses in order to minimize artifact contamination such as eye movement. To determine MEF1, we used the root mean square (RMS), which was calculated from the averaged waveforms of all MEG channels used for analyses (Figure 1A). We defined the peak amplitude of MEF1 as the amplitude of MEF1 and the peak latency as the latency of MEF1. We confirmed a clear dipole pattern in the contralateral central region at the peak latency of MEF1 in all subjects (Figure 1B).

We also performed dipole source analyses in the peak latency of MEF1 using free software for estimating cortical currents from MEG data (VBMEG; ATR Neural Information Analysis Laboratories, Kyoto, Japan) (Sato et al., 2004; Yoshioka et al., 2008; Toda et al., 2011; Yoshimura et al., 2012). Using each subject’s MRI data that was spatially normalized to the Montreal Neurological Institute (MNI) coordinates (Montreal, QC, Canada) using SPM5 (Wellcome Department of Cognitive Neurology, UK)^1^, dipole locations of MEFI were calculated on common MNI coordinates. To map current dipoles on the cortical surface, a polygon model of the cortical surface was constructed based on T1-weighted MRI data using FreeSurfer software (Martinos Center software)^2^ (Dale et al., 1999; Fischl et al., 1999, 2002). We defined the location on the MNI coordinates of strongest dipole moment around the left central sulci as the dipole location of MEF1 and that of the dipole moment value as the dipole intensity of MEF1 (Figure 1C).

In order to evaluate differences in MEF1 between genders, the difference in the average value between males and females was statistically analyzed for the four features of MEFI: latency, amplitude, dipole location, and dipole intensity. To evaluate the differences attributed to age, we sampled the pairs of all the combination from all 47 subjects in such a way that the age difference does not exceed 5 years and computed the correlation coefficients between difference of age and the features of MEF1. Differences in latency, amplitude, and dipole source characteristics (dipole location and intensity) of MEFI were computed for all MZ twin pairs and unrelated age-matched pairs. Unrelated pairs were made by randomly selecting 16 pairs from all subjects without any overlap in pair assignment. Pairs were made such that the age difference may be no more than 5 years.

## Results

Movement-evoked field 1 was consistently observed over the contralateral central region in all subjects. The mean latency of MEF1 was 113.0 ± 11.5 ms (mean ± SD), and the mean amplitude was 45.5 ± 18.9 fT/cm. Maximum values of individual differences in latency and amplitude of MEF1 were 48 ms and 78.7 fT/cm, respectively. Mean dipole location at the peak latency of MEF1 was (−45.3 ± 5.5, −17.7 ± 5.7, 60.2 ± 6.2) (MNI coordinates), and mean dipole intensity was 18.9 ± 13.4 nAm. There was no statistically significant difference among genders in latency, amplitude, dipole location, and dipole intensity of MEF1 (Table 1). All subjects were therefore analyzed independent of gender.

**Table 1:** **Gender differences in MEF1 components**.

MEF1 components	Male	Female	p*
Latency (ms)	113.4 (9.4)	113.0 (13.9)	0.903
Amplitude (fT/cm)	50.2 (22.2)	42.9 (13.4)	0.193
*x* coordinate	-45.8 (6.2)	-44.9 (6.6)	0.631
Dipole location *y* coordinate	-18.0 (4.8)	-17.5 (7.1)	0.756
*z* coordinate	58.2 (7.8)	61.7 (5.6)	0.093
Dipole intensity (nAm)	18.2 (13.1)	20.6 (14.6)	0.569

Values are indicated as means (standard deviation). *p Values of Student’s *t*-test between male and female subjects.

**Figure 2:**
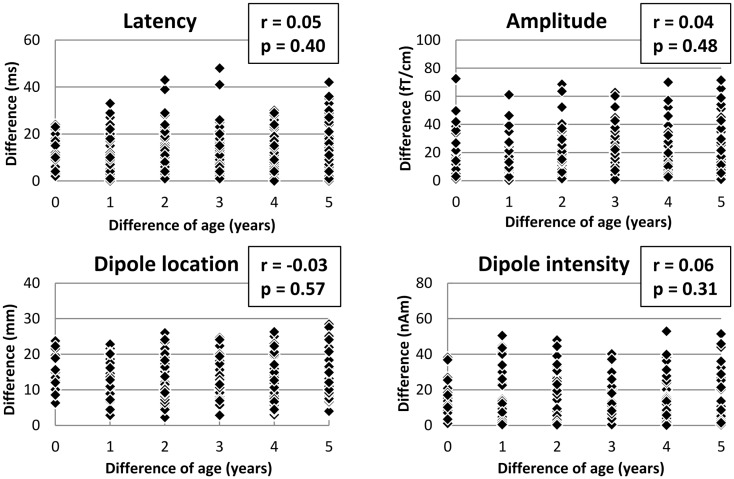
**Scatter diagrams showing the correlation between difference of age and the features of MEF1 in pairs of all combination (312 pairs)**. The unrelated pairs were selected in such a way that the age difference did not exceed 5 years. There were no significant correlations between difference of age and each feature (latency, amplitude, dipole moment, dipole intensity) (*p* < 0.05; Pearson’s correlation test).

Figure 2 shows the relationship between difference of age and difference of the features of MEF1 in pairs of all combinations of 47 subjects (312 pairs) such that the age difference may be no more than 5 years. Correlation coefficients for difference of age and difference of the latency, amplitude, dipole location, and dipole intensity were 0.05 (*p* = 0.40; Pearson’s correlation test), 0.04 (*p* = 0.71), −0.03 (*p* = 0.57), and 0.06 (*p* = 0.31), respectively. No significant correlation was observed in any features.

**Figure 3:**
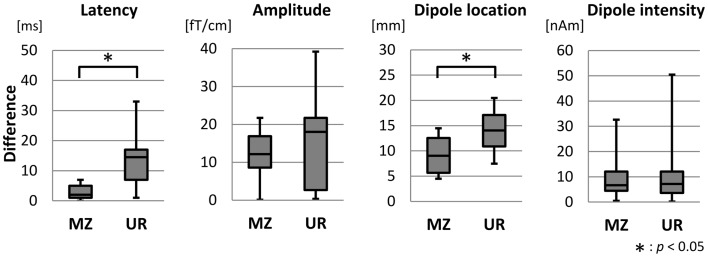
**Box plot diagrams showing the distribution of the features of MEF1 between MZ twin pairs and unrelated pairs**. The lowest and highest lines indicate the lowest and highest data values, respectively. The three lines that form the box indicate 25th, 50th, and 75th percentiles. Differences in the latency and the dipole location of MZ twin pairs were significantly smaller than that between unrelated pairs (Mann–Whitney *U* test, *p* < 0.05).

Figure 3 shows box and whisker plots of the differences in the latency, amplitude, dipole location, and dipole intensity of MEF1 between MZ twins and unrelated pairs. The average age of all 16 MZ twins was 62.2 ± 11.1 years, and the average age of the corresponding 16 unrelated pairs was 62.8 ± 10.2 years. Median values for differences in latency, amplitude, dipole location, and dipole intensity was 2.0 and 14.5 ms, 12.2 and 18.0 fT/cm, 9.0 and 14.0 mm, and 6.7 and 7.2 nAm in MZ twin pairs and unrelated pairs, respectively. Differences in the latency and dipole location among MZ twins were significantly less than those among unrelated pairs (Mann–Whitney *U* test, *p* < 0.05). However, there were no significant differences in the amplitude and dipole intensity among MZ twin pairs and unrelated pairs.

## Discussion

In this study, we found similarities in the latency and dipole location of MEF1 in MZ twins compared with unrelated pairs. These results indicate that genetic factors and early common environmental factors are dominant for these features of MEF1. Here, we discuss the similarities between MZ twins, particularly factors related to individual differences in the features of MEF1.

The latency of MEF1 in our study showed individual differences of up to 50 ms. A previous study reported that peak latencies of MEF1 ranged from 73.6 to 114 ms (Onishi et al., 2006). The difference among individuals was up to 40.4 ms, which was very similar to the results of our study. In addition, differences of the latency within MZ twins were significantly less than that of unrelated pairs, which means that the latency of MEF1 was similar in MZ twins. Because the average age of MZ twins in this study was >60 years, we assume that long-term differences in the living environments experienced after the MZ twins grew up and lived separately may have caused differences in their brain function. Nevertheless, homogeneity of MEFI latency was preserved even after long-term exposure to different environmental factors. This result indicates that the latency of MEF1 may be strongly affected by genetic and early common environmental factors. Alternatively, MEF1 latency may be affected by nerve conduction velocity, which may reflect myelination. The process of myelination begins early in fetal development and continues systematically for several years (Yakovlev, 1962). In particular, the sensorimotor cortex is involved in the primary myelogenic area, where myelination proceeds from early brain development. Thus, myelination in the sensorimotor cortex may be influenced by genetic and early common environmental factors, and may contribute to the homogeneity of MEF1 latency in the present study. We also found homogeneity in the dipole location in MZ twins. Previous twin studies using MRI reported that strong anatomical similarities were observed in frontal and parietal lobes in MZ twins (Eyler et al., 2011, 2012). Taken together, anatomical homogeneity in the frontoparietal area between twins may lead to the similarity of dipole location. Unlike the latency and dipole location of MEF1, we found no significant similarity between amplitude and dipole intensity within MZ twins. The lack of difference in amplitude and dipole intensity between twins and unrelated pairs was likely not a result of the varied movement intensity of individual subjects, but rather that amplitude and dipole intensity of MEF1 are affected by different environmental factors. In previous studies, it has been reported that amplitude and dipole moment of MEF1 does not change even if finger movement intensity and frequency are changed (Mayville et al., 2005; Onishi et al., 2006). In addition, Van’t Ent et al. (2010) reported that amplitude of somatosensory-evoked magnetic field (SEF) is influenced by genetics. SEF induced by electrical nerve stimulation reflects peripheral afferents as well as MEF1. However, SEF reflects cutaneous afferents (Kakigi et al., 2000) while MEF1 reflects sensory feedback from muscle spindle receptors induced by muscle contraction (Oishi et al., 2004). Therefore, physiological differences in the peripheral receptors may be the reason for the difference between MEF1 and SEF. Although individual motor performance was not evaluated in details in the present study, the sensitivity of muscle spindle changes by strength training (Hakkinen and Komi, 1983). Taken together, it is suggested that the amplitude of the evoked field from muscle spindles show plasticity and is likely influenced by the individual environmental factors. Specifically, we have demonstrated that MEF1 latency and dipole location are affected by genetic and early common environmental influence, whereas MEF1 amplitude and dipole intensity are affected by long-term individual environmental influences.

The present study is limited to comparisons of MRCFs between MZ and unrelated pairs instead of comparisons between MZ and DZ, which would be ideal for discriminating between genetic and environmental influences. Comparisons of MZ with unrelated pairs fail to decipher genetic from early common environmental influences. However, because middle-aged and elderly subjects have been exposed to individual adulthood environments for sufficiently long times to allow discrimination of these effects from those of genetic and early common environmental influences.

## Conclusion

We found that the latency and the current source location of MEF1 were more homogenous between middle-aged and elderly MZ twins than between unrelated pairs, whereas such homogeneity was not found in the amplitude and the current source intensity of MEF1. These results suggest that some of the basic neurophysiological factors of cerebral motor function are determined early in life by genetic and early common environmental factors, while others are influenced by long-term environmental factors. These data improve the understanding of genetic and environmental factors that influence cerebral motor function and may contribute to assessments of individual motor function.

## Conflict of Interest Statement

The authors declare that the research was conducted in the absence of any commercial or financial relationships that could be construed as a potential conflict of interest.

## Footnotes

^1^http://www.fil.ion.ucl.ac.uk/spm

^2^http://surfer.nmr.mgh.harvard.edu/
